# A Gene-Based Positive Selection Detection Approach to Identify Vaccine Candidates Using *Toxoplasma gondii* as a Test Case Protozoan Pathogen

**DOI:** 10.3389/fgene.2018.00332

**Published:** 2018-08-20

**Authors:** Stephen J. Goodswen, Paul J. Kennedy, John T. Ellis

**Affiliations:** ^1^School of Life Sciences, University of Technology Sydney, Ultimo, NSW, Australia; ^2^School of Software, Faculty of Engineering and Information Technology, Centre for Artificial Intelligence, University of Technology Sydney, Ultimo, NSW, Australia

**Keywords:** *Toxoplasma gondii*, Hammondia hammondi, Neospora caninum, reverse vaccinology, vaccine discovery, positive selection

## Abstract

Over the last two decades, various *in silico* approaches have been developed and refined that attempt to identify protein and/or peptide vaccines candidates from informative signals encoded in protein sequences of a target pathogen. As to date, no signal has been identified that clearly indicates a protein will effectively contribute to a protective immune response in a host. The premise for this study is that proteins under positive selection from the immune system are more likely suitable vaccine candidates than proteins exposed to other selection pressures. Furthermore, our expectation is that protein sequence regions encoding major histocompatibility complexes (MHC) binding peptides will contain consecutive positive selection sites. Using freely available data and bioinformatic tools, we present a high-throughput approach through a pipeline that predicts positive selection sites, protein subcellular locations, and sequence locations of medium to high T-Cell MHC class I binding peptides. Positive selection sites are estimated from a sequence alignment by comparing rates of synonymous (dS) and non-synonymous (dN) substitutions among protein coding sequences of orthologous genes in a phylogeny. The main pipeline output is a list of protein vaccine candidates predicted to be naturally exposed to the immune system and containing sites under positive selection. Candidates are ranked with respect to the number of consecutive sites located on protein sequence regions encoding MHCI-binding peptides. Results are constrained by the reliability of prediction programs and quality of input data. Protein sequences from *Toxoplasma gondii* ME49 strain (TGME49) were used as a case study. Surface antigen (SAG), dense granules (GRA), microneme (MIC), and rhoptry (ROP) proteins are considered worthy *T. gondii* candidates. Given 8263 TGME49 protein sequences processed anonymously, the top 10 predicted candidates were all worthy candidates. In particular, the top ten included ROP5 and ROP18, which are *T. gondii* virulence determinants. The chance of randomly selecting a ROP protein was 0.2% given 8263 sequences. We conclude that the approach described is a valuable addition to other *in silico* approaches to identify vaccines candidates worthy of laboratory validation and could be adapted for other apicomplexan parasite species (with appropriate data).

## Introduction

Since the inception of reverse vaccinology (Rappuoli, 2000) almost two decades ago, researchers have applied various *in silico* approaches to identify protein and/or peptide vaccines candidates worthy of laboratory validation. These approaches were previously reviewed (Bowman et al., 2011; Jones, 2012; Donati and Rappuoli, 2013; Rappuoli et al., 2016). Fundamental to each approach is the detection of signals or patterns encoded in protein sequences of a target pathogen. As to date, no signal has been identified that clearly indicates a protein will effectively contribute to a protective immune response in a host. Consequently, the current best practice is to predict pertinent protein characteristics from informative signals that collectively support the likelihood the protein will make a credible candidate. Although there is no proven set of characteristics, the general community consensus delineating a valued characteristic is one inferring that a protein is either external to or located on, or in, the membrane of a pathogen. Such a protein type is deemed more accessible to surveillance by the immune system than one within the interior of a pathogen (Davies and Flower, 2007; Flower et al., 2010). In this study, we investigate the amino acid substitution rate of a protein as an additional characteristic supporting its vaccine candidacy.

Protein sequences from *Toxoplasma gondii* were used as a case study. *Toxoplasma* is an obligate intracellular pathogen responsible for birth defects in humans (Montoya and Liesenfeld, 2004) and an important model system for the phylum Apicomplexa (Roos et al., 1999; Kim and Weiss, 2004; Che et al., 2010). An apicomplexan pathogen invades a host cell first by, recognizing host-cell surface receptors via surface antigens (SAGs) on its cell membrane, and then secreting proteins (excreted/secreted antigens) from the organelles of apical complexes, including Dense Granules (GRA), Micronemes (MIC), and Rhoptries (ROP) (Chen et al., 2008). Consequently, SAG, GRA, MIC and ROP proteins have been the primary antigens under investigation in numerous recombinant/subunit vaccine studies (Kur et al., 2009; Zhang et al., 2015) due to their natural exposure to the immune system and potential to induce a host immune response. These latter proteins are referred to henceforth as target candidates.

All pathogen proteins are susceptible to various types of selection in response to environmental pressures. This is from the perception that natural selection acts mostly on expressed proteins (i.e., the phenotype) rather than directly on genetic material. The three main types of selection are positive, negative/purifying, and balancing (Harris and Meyer, 2006; Oleksyk et al., 2010). The selection type of interest in this study is positive with the main environmental pressure being the immune system. We use the term ‘positive selection’ in the context of any type of selection where newly derived mutation has a selective advantage over other mutations and that the majority of the fixed mutations are adaptive even if most mutations are deleterious or neutral (Kaplan et al., 1989; Thiltgen et al., 2017).

Pathogens are believed to represent one of the strongest selective pressures acting on humans (Fumagalli et al., 2011). Conversely, pathogens are naturally under strong selection to prevent detection from the host’s immune system. Eventually, advantageous escape mutations become fixed at the molecular level. However, the host’s immune system is also under strong selection for mutations that enable the pathogen to be detected. Subsequently, once again, the pathogen will experience selection for new escape mutations in this evolutionary arms race (Thiltgen et al., 2017). This see-sawing process leaves sequence patterns of variation indicating selected and neutral regions, i.e., genomic footprints known as selection signatures. Proteins that continually avoid detection by the immune system are expected not to have these signatures.

The premise for this study was that proteins under positive selection, as identified by selection signatures, are more likely suitable vaccine candidates than proteins under negative or purifying selection. The expectation is that those proteins naturally exposed to the immune system, such as the target candidates, will possess and maintain higher levels of genetic variation than interior ones; in effect creating a greater pool of mutations for natural selection to act upon to avoid recognition by the immune system (Pacheco et al., 2012; Obara et al., 2016; Bigham et al., 2018; Garzon-Ospina et al., 2018).

One of the best known gene-based methods to detect positive selection at the molecular level is based on codon analysis. This method compares patterns of synonymous and non-synonymous mutations in protein coding sequences from divergent species. Synonymous mutations (functionally silent or neutral) are presumed not to change the amino acid sequence of the protein encoded, whereas non-synonymous mutations do alter the amino acid sequence and are subject to natural selection. The synonymous substitution rate is the neutral rate μ_S_ = μ. In contrast, the non-synonymous substitution rate will be typically different to the neutral rate μ_N_ ≠ μ.

Ideally, we need a chronological series of ancestor genes to truly calculate μ_N_ and μ_S_ rates. This is obviously an impracticable ideal and so non-synonymous and synonymous distances, among coding sequences of orthologous genes in a phylogeny, are estimated from a sequence alignment (*dN* = *t*μ_N_ and *dS* = *t*μ*_S_* where *t* is the time of divergence or branch length in the phylogeny). The substitution rate ratio (ω) is equal to dN/dS. This ratio quantifies the strength and denotes the type of molecular selection pressures acting on protein-coding regions (Kryazhimskiy and Plotkin, 2008). Most proteins are observed to be under purifying selection. However, a continued elevated rate of amino acid change in some proteins is expected as a consequence of pathogen-host co-evolution (Thiltgen et al., 2017). A review (Kryazhimskiy and Plotkin, 2008) explains the relationship between selection and dN/dS over long time-scales.

Epitopes (short peptides) are the minimal structure recognized by the immune system and are the principal components of subunit vaccines (Korber et al., 2006). More specifically, it is the recognition of epitopes by T- and B-cells (and soluble antibodies) that activates the cellular and humoral immune response (Flower et al., 2010). Several studies have shown that protective immunity to *T. gondii* is through cell-mediated responses (Denkers and Gazzinelli, 1998; Innes et al., 2002; Williams and Trees, 2006; Dlugonska, 2008).

T-cell epitopes, which are typically short linear peptides, are derived from pathogen or host proteins (Hanada et al., 2004). These peptides are bound by major histocompatibility complexes (MHC) and presented by antigen-presenting cells (APCs) for inspection by T-cell receptors (TCRs) (Korber et al., 2006). Recognition of peptides by TCRs on CD8+ T cells cause the secretion of IFN-γ, which activates macrophages to inhibit replication, kill the parasite, and induce lysis of infected cells (Cong et al., 2011). Identifying proteins that encode MHC Class I (MHCI) restricted peptides is of interest because CD8+ T cells recognize epitopes presented in the context of MHCI molecules. Furthermore, the expectation here is that protein sequence regions from which these peptides originated will contain signatures of selection. Studies support that different regions of a protein, are potentially subject to different selective pressures, particularly regions of functional relevance (Jin et al., 2012; Vitti et al., 2013) and notably, in regions encoding T-cell epitopes (Hughes, 1991). This study uses a T-Cell MHC class I binding program to predict the physical location of medium to high binding peptides on *T. gondii* proteins identified to have positive selection sites.

We now present an approach that first predicts the *T. gondii* proteins that are naturally exposed to the immune system and contain sites under positive selection; and then rank these candidates with respect to the number of consecutive sites located on epitopes. Our aspiration was to take anonymous *T. gondii* protein sequences and predict the target candidates. The expectation is that the described approach can be adapted for other apicomplexan parasite species with appropriate data.

## Materials and Methods

### Data Collection

Protein and mRNA sequences in a FASTA format were downloaded from EupathDB (Aurrecoechea et al., 2010) for the Apicomplexan species listed in **Table 1**. All downloaded protein sequences were validated to ensure that they commenced with the letter M and did not contain invalid letters, e.g., J, O, U, and X. All mRNA sequences were validated to ensure that they commenced with ATG; terminated with TGA, TAA, or TAG; contained only letters A, T, G, and C; and their sequence lengths were a multiple of three for later codon analysis. Furthermore, the mRNA sequences were checked to confirm that their codon translations matched their corresponding protein sequences. The related mRNA and protein sequences were then classified into three datasets named Species 16, Species 25, and Species 55 in accordance to how many species (or strains) were assigned to the dataset. The datasets were based on published phylogenetic relationships (Kuo et al., 2008; Morrison, 2009). Our mindset was to evaluate whether introducing more distantly related sequences to the potential ortholog groups increased CODEML’s power to accurately estimate dN and dS. That is, the 25 and 55 species datasets incrementally introduced more distantly related species to the core 16 species dataset containing the target species.

**Table 1:** List of Apicomplexan species used in study.

Data group	Species^a^
16 species	*Hammondia hammondi* strain H.H.34 (HHA:8007), *Neospora caninum* Liverpool (NCLIV:7131), *Sarcocystis neurona* SN3 (SN3:6965), *Sarcocystis neurona* SO SN1 (SCRN:7077), *Toxoplasma gondii* ARI (TGARI:9958), *Toxoplasma gondii* FOU (TGFOU:10117), *Toxoplasma gondii* GAB2-2007-GAL-DOM2 (TGDOM2:9136), *Toxoplasma gondii* GT1 (TGGT1:8460), *Toxoplasma gondii* MAS (TGMAS:10005), ***Toxoplasma gondii* ME49 (TGME49:8322)**, *Toxoplasma gondii* RH (TogoCp:26), *Toxoplasma gondii* RUB (TGRUB:10027), *Toxoplasma gondii* TgCatPRC2 (TGPRC2:10121), *Toxoplasma gondii* VAND (TGVAND:9255), *Toxoplasma gondii* VEG (TGVEG:8410), *Toxoplasma gondii* p89 (TGP89:9701).
25 species	14 Set + *Cyclospora cayetanensis* strain CHN_HEN01, *Eimeria acervulina* Houghton, *Eimeria brunetti* Houghton, *Eimeria falciformis* Bayer Haberkorn 1970, *Eimeria maxima* Weybridge, *Eimeria mitis* Houghton, *Eimeria necatrix* Houghton, *Eimeria praecox* Houghton, *Eimeria tenella* strain Houghton.
55 species	14 and 25 Set + *Cryptosporidium andersoni* isolate 30847, Cryptosporidium hominis TU502, *Cryptosporidium hominis* UdeA01, *Cryptosporidium hominis* isolate TU502_2012, *Cryptosporidium muris* RN66, *Cryptosporidium parvum* Iowa II, *Cryptosporidium ubiquitum* isolate 39726 *Gregarina niphandrodes* Unknown strain, *Plasmodium berghei* ANKA, *Plasmodium chabaudi* chabaudi, *Plasmodium coatneyi* Hackeri, *Plasmodium cynomolgi* strain B, *Plasmodium falciparum* 3D7, *Plasmodium falciparum* IT, *Plasmodium fragile* strain nilgiri, *Plasmodium gaboni* strain SY75, *Plasmodium gallinaceum* 8A, *Plasmodium inui* San Antonio 1, *Plasmodium knowlesi* strain H, *Plasmodium malariae* UG01, *Plasmodium ovale curtis*i GH01, *Plasmodium reichenowi* CDC, *Plasmodium relictum SGS1-like*, *Plasmodium vinckei* petteri strain CR, *Plasmodium vinckei vinckei* strain vinckei, *Plasmodium vivax* P01, *Plasmodium vivax* Sal-1, *Plasmodium yoelii yoelii* 17X, *Plasmodium yoelii yoelii* 17XNL, *Plasmodium yoelii yoelii* YM.

*^*a*^Target species highlighted in bold. The protein prefix identifier and the number of proteins are contained in brackets for the 16 species dataset.*

### Data Workflow for Predicting Positive Selection Sites

**FIGURE 1:**
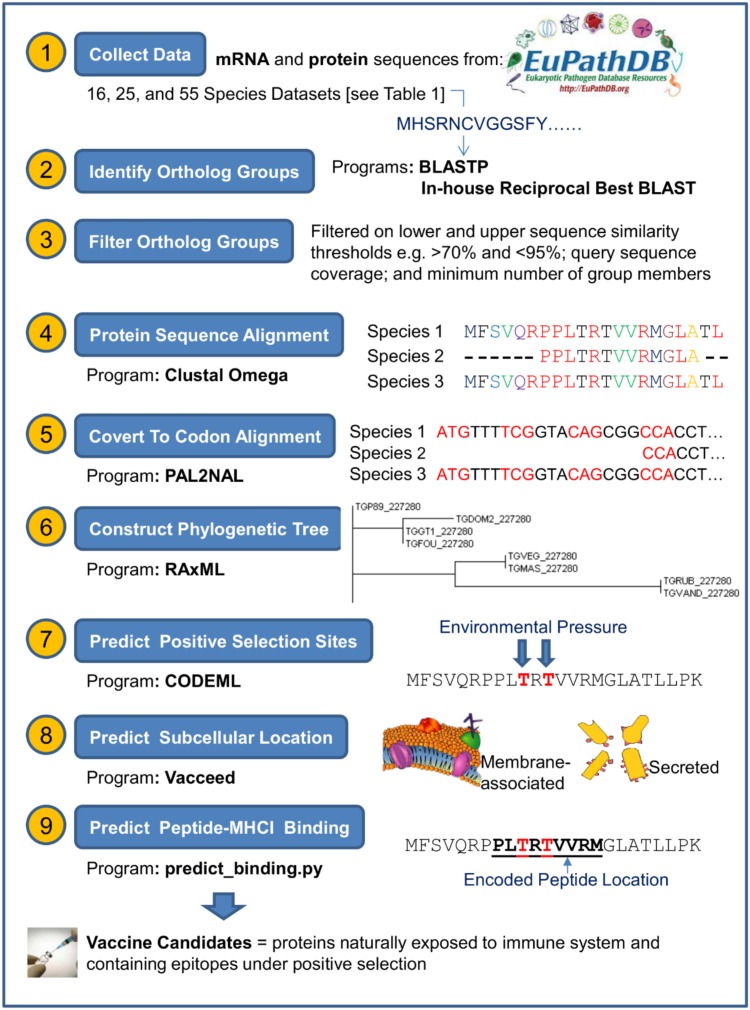
Schematic of steps taken to predict vaccine candidates given proteins sequences from a target pathogen. This figure represents an overview of the steps and programs used to ultimately classify those proteins that are naturally exposed to the immune system and contain regions encoding major histocompatibility complexes (MHC) binding peptides under positive selection. Further program details are shown in **Table 2** and **Supplementary Table S1**.

The three datasets were processed independently in an in-house pipeline that linked the input and output of the programs listed in **Table 2**. **Supplementary Table S1** shows the key command-line syntax for these programs. Our pipeline and methodology was adapted and extended from programs and methods proposed by Jeffares et al. (2015). The overall initiative of the pipeline was to generate the appropriate input files for CODEML, which are: (1) a codon-based alignment of the DNA sequences from ortholog group members and, (2) a phylogenetic tree of these members. The pipeline steps to generate these files are now described. **Figure 1** represents a schematic of the pipeline data workflow. *T. gondii* strain ME49 (referred to here as TGME49) was used as the target species to test and establish the most appropriate workflow/pipeline to be adapted for other apicomplexan data, such as from *Neospora caninum* strain Liverpool (NCLIV) and *Hammondia hammondi* strain H.H.34 (HHA).

**Table 2:** Programs used in the study [download date: July 2017].

Program	Version	^a^Function	Download URL	Reference
^b^BLASTP	2.6.0	Performs a protein vs. protein sequence alignment	ftp://ftp.ncbi.nlm.nih.gov/blast/executables/blast+/LATEST/	Camacho et al., 2009
Clustal Omega	1.2.4	Computes a multiple sequence alignment	http://www.clustal.org/omega/	Sievers et al., 2011
PAL2NAL	14	Converts a multiple sequence alignment of proteins and the corresponding mRNA sequences into a codon-based DNA alignment.	http://www.bork.embl.de/pal2nal/#Download	Suyama et al., 2006
RAxML	8.2.10	Creates a phylogenetic tree based on maximum-likelihood inference.	https://github.com/stamatak/standard-RAxML	Stamatakis, 2014
^c^CODEML	4.9e	Computes substitution rate ratio (dN/dS)	http://abacus.gene.ucl.ac.uk/software/paml.html	Yang, 2007
^d^predict_binding.py	2.17	Predicts peptides binding to Major Histocompatibility Complex (MHC) class I molecules	http://tools.iedb.org/mhci/download/	Kim et al., 2012
WoLF PSORT	0.2	Predicts subcellular localization sites of proteins	^e^https://wolfpsort.hgc.jp	Horton et al., 2007
SignalP	4.1	Predicts the presence and location of signal peptide cleavage sites	http://www.cbs.dtu.dk/services/SignalP/	Petersen et al., 2011
TargetP	1.1	Predicts subcellular location	http://www.cbs.dtu.dk/services/TargetP/	Emanuelsson et al., 2007
TMHMM	2.0	Predicts transmembrane helices	http://www.cbs.dtu.dk/services/TMHMM/	Krogh et al., 2001
Phobius	1.01	A combined transmembrane topology and signal peptide predictor	http://phobius.sbc.su.se/	Käll et al., 2004
Vacceed	1.0	Predicts secreted and/or membrane-associated proteins	https://github.com/sgoodswe/vacceed	Goodswen et al., 2014


*^*a*^The specific function utilized by this study (other functions can be performed).****^*b*^BLASTP****is a program within the****BLAST+****package (a NCBI Basic Local Alignment Search Tool).****^*c*^CODEML****is a program within the****PAML****package (a suite of programs for model fitting and phylogenetic tree reconstruction using nucleotide or amino-acid sequence data). ^*d*^predict_binding.py is the frontend to a collection of prediction methods. The methods used by the study are ann, smm, and netmhcpan. ^*e*^Contact program creators for standalone version.*

#### Step 1: Identify Ortholog Groups

BLASTP was performed between the protein sequences of the different species or strains within each dataset. For example, the protein sequences from TGME49 were aligned in turn with the sequences from HHA, then NCLIV and so on. In effect, TGME49 sequences aligned with sequences from 15, 24 and 54 other species with respect to the dataset. An in-house python script processed the BLASTP output and executed a Reciprocal Best BLAST hit (RBH) method (Moreno-Hagelsieb and Latimer, 2008; Salichos and Rokas, 2011) to determine ortholog groups. This method essentially works as follows: given protein A from TGME49, protein B from NCLIV and protein C from HHA – for these proteins to be in the same ortholog group; protein B must be the best BLASTP hit to protein A, and protein C must be the best hit to either protein A or protein B.

#### Step 2: Filter Ortholog Groups

Three separate sets of filtered groups were generated and ran independently through the remaining pipeline. That is, the ortholog groups were filtered such that each member of the three filtered groups either had less than 90, 95, or 99% protein sequence similarity, respectively. A further group membership requirement was a greater than 70% sequence similarity and greater than 70% query coverage (i.e., the percent that a BLASTP query sequence aligns to a target sequence). Additionally, each filtered group contained only one protein per species (or strain). That is, paralogs were excluded to remove their computational complication when estimating dN and dS (Jeffares et al., 2015). Any filtered group with less than five members were ignored from further processing (the PAML documentation recommends that the absolute minimum is 4 or 5 if the sequence divergence is optimal). Randomly selected ortholog groups were compared to manually curated groups contained in OrthoMCL (a database of ortholog groups of proteins) (Li et al., 2003). OrthoMCL does not facilitate for high-throughput ortholog group verification, but our comparisons provided a general indication that the RBH script to generate the orthologs was correct. Groups meeting the requirements were processed through the ongoing pipeline as separate entities. That is, a filtered ortholog group is independent of any other group.

#### Step 3: Preform a Sequence Alignment of Ortholog Group Members

The protein sequences associated with the ortholog group members were aligned using Clustal Omega.

#### Step 4: Convert to Codon Alignments

PAL2NAL was used to convert the protein sequence alignments of each ortholog group into corresponding codon alignments. An important PAL2NAL parameter is ‘-nogap.’ This is because an alignment gap in CODEML is treated as an undetermined nucleotide and is removed from the analysis. Removal of any nucleotide means removal of the whole codon.

#### Step 5: Construct Phylogenetic Tree

A phylogenetic tree for each ortholog group was constructed using RAxML. This program makes available a wide range of user parameter settings. Two important parameters set the type of algorithm and DNA substitution model. The algorithm selected for this study conducted a rapid Bootstrap analysis and searched for the best-scoring maximum likelihood tree in one single program run. The selected substitution model was generalized time reversible (GTR) GAMMA (Yang, 1994). **Supplementary Table S1** shows the program parameter settings used to construct the tree.

#### Step 6: Compute Substitution Rate Ratio (ω)

**FIGURE 2:**
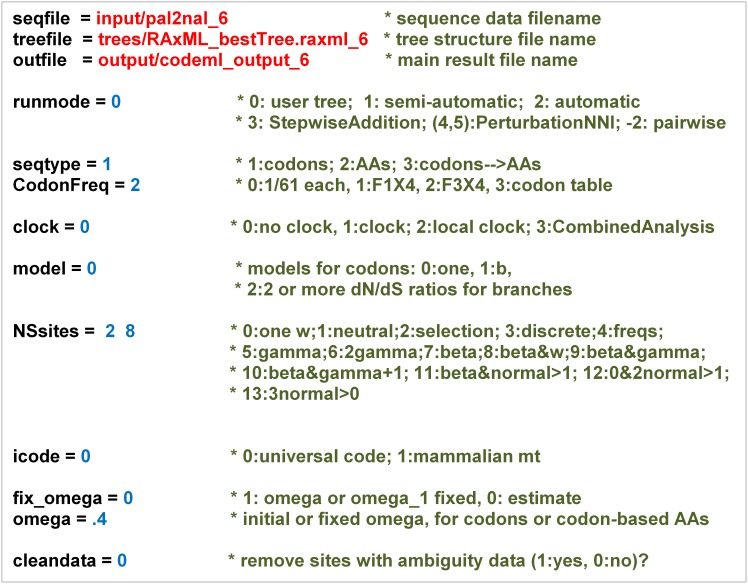
CODEML configuration file settings used to estimate the amino acid sites under positive selection. This figure shows the values set within the CODEML configuration file to detect positive selection sites in target pathogen proteins. CODEML is a program within the Phylogenetic Analysis by Maximum Likelihood (PAML) package that estimates non-synonymous and synonymous evolutionary rates. Configuration values set by a user follow the ‘=’. The same settings were used for each processed ortholog group – except seqfile, treefile, and outfile were changed to uniquely match a specific group. Text preceding with a ‘^∗^’ is a comment, which specifies the range of possible options. These options are explained in detail in the PAML Manual (http://abacus.gene.ucl.ac.uk/software/paml.html).

The CODEML program within the Phylogenetic Analysis by Maximum Likelihood (PAML) package uses maximum likelihood to statistically estimate dN and dS. It uses the observed changes present in the codon alignments from PAL2NAL, given the phylogenetic tree constructed by RAxML. CODEML calculates the likelihood of the observed changes resulting from two models of evolution, only one of which allows for the possibility of detecting positive selection (dN/dS > 1). **Figure 2** shows the values set within the CODEML configuration file for this study, including the site models (NSsites) setting to test ω varying at different sites.

### Interpretation of Positive Selection Results

The dN/dS ratio can indicate one of three types: (1) neutral evolution, when an amino acid change is fixed at the same rate as a synonymous mutation (dN/dS = 1); (2) positive (Darwinian, directional/adaptive or diversifying) selection, when an advantageous amino acid change is fixed at a higher rate than a synonymous mutation (dN/dS > 1); and (3) purifying (negative or background) selection, when a deleterious amino acid change reduces its fixation rate (dN/dS < 1). For CODEML, the main results of interest to this study are under the Bayes Empirical Bayes (BEB) analysis (Yang et al., 2005) section of the CODEML output (BEB is only computed with NSsite models 2 and 8). This section lists the location and the posterior probability of positively selected sites (i.e., where dN/dS > 1) on the target protein. Significant sites with posterior probability > 99% are designated with ‘^∗∗^’ and sites with posterior probability > 95% but <or =to 99% designated with ‘^∗^’. The total number of sites under positive selection for each *T. gondii* protein in the ortholog groups were recorded along with the number of significant sites (i.e., the sites designated with ‘^∗^’ or ‘^∗∗^’).

### Peptide-MHC I Binding Predictions

A Linux standalone version of a T-Cell MHC class I binding predictor (named predict_binding.py) was used to predict binding peptides. This predictor was downloaded from the Immune Epitope Database and analysis resource (IEDB) (Kim et al., 2012). The protein sequence for every *T. gondii* protein from the filtered ortholog groups (see step one in section “Data Workflow for Predicting Positive Selection Sites”) was input into the peptide-MHC binding predictor. There are thousands of known MHC class I alleles. However, we predicted peptides binding to 76 common human MHCI alleles (in effect, each protein was tested against 304 MHC allele-peptide length combinations. MHCI binds to peptides that are typically eight to eleven amino acid residues in length. Therefore, 76 alleles ^∗^ 4 peptide lengths = 304 combinations. ‘Common’ in this instance refers to alleles that occur in at least 1% of the human population or have an allele frequency of 1% or higher). The affinity of an MHC allele and binding peptide is deemed greater the lower the IC_50_ nM score. The predictor developers’ propose a rough guideline for interpretation of the score: peptides with IC_50_ values <50 nM are considered high affinity, <500 nM intermediate affinity, and <5000 nM low affinity. The predicted sites under positive selection were mapped to the intermediate and high affinity peptides using an in-house Python script.

Published epitopes related to TGME49 were also downloaded from IEDB in a comma-separated values (CSV) format. The selected online search filters were ‘Linear Epitopes,’ *T. gondii* ME49 for ‘Antigen,’ ‘All assay choices’ selected, ‘Any MHC Restriction,’ ‘Any Host,’ ‘Any Disease,’ and ‘Any Reference Type.’ The number of epitopes downloaded was 110. These epitopes were mapped to TGME49 gene identifiers and are listed in **Supplementary Table S2**.

### Predicting Proteins Naturally Exposed to the Immune System

The following programs were used to predict if the target proteins (e.g., TGME49, NCLIV, and HHA) were secreted or membrane associated, i.e., naturally exposed to the immune system: SignalP 4.0 (Petersen et al., 2011) (predicts presence and location of signal peptide cleavage sites); WoLF PSORT 0.2 (Horton et al., 2007) and TargetP 1.1 (Emanuelsson et al., 2007) (predict subcellular localization); TMHMM 2.0 (Krogh et al., 2001) (predicts transmembrane domains in proteins); Phobius (Kall et al., 2004) (predicts transmembrane topology and signal peptides); and Vacceed (Goodswen et al., 2014) (computes the probability that a protein is naturally exposed to the immune system). These programs were essentially chosen because they are applicable to eukaryotes, could be freely downloaded, run in a standalone mode, allow high throughput processing, and execute in a Linux environment. The threshold values applied to program outputs for exposed (e.g., secreted or membrane-associated) classification are SignalP ≥ 0.6 (secreted); WoLF PSORT = ‘membrane,’ ‘secreted’ or ‘membrane_and_secreted’; TargetP ≥ 0.6 (secreted); tmhmm_ExpAA ≥ 18 (membrane-associated, or secreted if tmhmm_First60 ≥ 10); tmhmm_First60 ≥ 10 (secreted); tmhmm_PredHel ≥ 0 (membrane); Phobius_TM ≥ 0 (membrane), Phobius_SP = ‘Y’ (secreted), and Vacceed ≥ 0.5 (where ‘≥’ denotes greater than or equal to’).

## Results

A total of 8322 protein sequences along with their originating mRNA sequences were downloaded from EupathDB for the test case target species, *T. gondii* ME49. These sequences were validated as per section 2.1 and 59 proteins were removed from further processing (i.e., a protein was removed if either the mRNA or protein sequence was invalid). **Supplementary Table S2** lists the invalid protein IDs and the reason(s) for removal.

Protein sequences from each of the 8263 valid TGME49 proteins were aligned in turn with the sequences from 15, 24 to 54 other species with respect to the dataset. Theoretically, if each of the 8263 sequences were to fulfill the sequence similarity and query coverage criteria with at least five other species (as per step two described in section “Data Workflow for Predicting Positive Selection Sites”), the maximum possible number of orthologs groups would be 8263. **Table 3** shows the actual number of filtered ortholog groups per dataset following step two. As expected the larger the upper similarity threshold, the more inclusive of closely related species and the greater the number of ortholog groups. Conversely, the smaller the upper similarity threshold, the smaller the number of ortholog groups. **Table 3** also illustrates the number of predicted candidates by the pipeline given the ortholog groups. A candidate, in this case, is a TGME49 protein that is predicted to be exposed to the immune system and contain sites under positive selection. More specifically, a predicted candidate has a Vacceed score ≥ 0.5 and a CODEML generated count of significant positive selection sites greater than zero. Some of these candidates are deemed true positives whilst others are false. A true positive is a target candidate. That is, those proteins with annotation that contain either the words ‘dense granule protein GRA,’ ‘microneme protein MIC,’ ‘rhoptry protein ROP,’ ‘SAG-related sequence SRS’ or ‘Toxoplasma gondii family’ as part of their protein name. **Supplementary Table S2** lists the 244 out of 8263 proteins that match the latter annotation: 16 proteins contain ‘dense granule protein GRA’ (16/8263 = 0.19% of TGME49 known proteins), 19 ‘microneme protein MIC’ (0.23%), 17 ‘rhoptry protein ROP’ (0.2%), 111 ‘SAG-related sequence SRS’ (1.34%) and 81 ‘Toxoplasma gondii family’ (0.98%). The ‘Toxoplasma gondii family’ proteins are categorized A to E, where there are 33 ‘A,’ 15 ‘B,’ 14 ‘C,’ 11 ‘D,’ and 8 ‘E’ annotated proteins.

**Table 3:** Number of ortholog groups per dataset and the number of predicted candidates.

Species dataset	Filter set	Dataset similarity criteria^a^	Ortholog groups		Number of candidates^d^
			Input^b^	Output^c^	
16	1	>70% and <99%	3139	2986	651 (280)
16	2	>70% and <95%	143	130	60 (43)
16	3	>70% and <90%	22	19	11 (8)
25	1	>70% and <99%	3606	3373	663 (290)
25	2	>70% and <95%	581	520	61 (44)
25	3	>70% and <90%	252	226	16 (13)
55	1	>70% and <99%	3522	NC	NC
55	2	>70% and <95%	597	527	63 (46)
55	3	>70% and <90%	314	288	17 (14)


**^*a*^Each member of the filtered ortholog group have a specific greater than or less than protein sequence similarity. Other criteria for group membership include 70% query coverage (the percent that the BLASTP query sequence aligned to the target sequence) and only one protein per species or strain. ^*b*^Number of ortholog groups input into pipeline for predicting positive selection sites. ^*c*^Number of ortholog groups output from pipeline, i.e., some input ortholog groups generated non-specific errors and subsequently output no (or unreliable) results. ^*d*^Number of predicted protein candidates for Toxoplasma gondii ME49, i.e., proteins predicted to be exposed to the immune system and contain positive selection sites. The number in brackets is the number of predicted candidates excluding hypothetical proteins. NC, Not Computed (pipeline terminated after 15 days of processing).**

**Table 4** shows the prediction outcomes in predicting a target candidate when given different species datasets. The sequence similarity criteria > 70 and <95 resulted in the best positive predictive value (PPV) for each of the species datasets. This clearly shows that including more closely or distantly related sequences limits the predictive power of the approach, i.e., >95 (more closely related) or <90 (more distantly related) results in lower PPV. Furthermore, the results show that including more species related to TGME49 (i.e., those in the 25 and 55 species datasets) did not improve the predictive power.

**Table 4:** Comparisons between predicted outcomes from different species datasets when predicting target candidates for *Toxoplasma gondii* ME49.

Species dataset^a^	Similarity criteria^b^	TP	FP	FN	TN	SP (%)	SN (%)	PPV (%)	NPV (%)	Processing time (hms)^c^
16	>70% and <99%	90	189	35	894	72	82	32	96	65 h 25 m 6 s
16	>70% and <95%	40	3	13	27	75	90	93	68	1 h 53 m 48 s
16	>70% and <90%	7	1	2	3	78	75	88	60	12 m 4 s
25	>70% and <99%	87	203	53	1257	62	86	30	96	97 h 3 m 51 s
25	>70% and <95%	38	6	14	389	73	98	86	96	8 h 46 m 43 s
25	>70% and <90%	6	7	3	192	67	96	46	98	5 h 1 m 8 s
55	>70% and <95%	40	6	12	400	76	98	87	97	360 h 14 m 24 s
55	>70% and <90%	8	6	3	247	73	98	27	99	202 h 26 m 14 s


*A predicted candidate is one that has a Vacceed score ≥ 0.5 and a CODEML generated count of significant positive selection sites > 0. The target candidates are proteins containing either the words ‘dense granule protein GRA,’ ‘microneme protein MIC,’ ‘rhoptry protein ROP,’ ‘SAG-related sequence SRS,’ or ‘Toxoplasma gondii family’ as part of their protein name (these proteins are naturally exposed to the immune system and expected to have sites under positive selection). TP = true positives = number of correctly predicted target candidates, FP = false positives = number of exposed proteins under positive selection but not recognized as target candidates; FN = false negatives = number of target candidates incorrectly predicted to be non-exposed and/or under negative or neutral selection; TN = true negatives = number of proteins correctly predicted to be non-exposed and under negative or neutral selection; SN = sensitivity = % of target candidates correctly predicted = TP/(TP + FN), SP = % of non-candidates correctly predicted = TN/(FP + TN); PPV = positive predictive value = % of target candidates that are true positives = TP/(TP + FP); NPV = negative predictive value = % of non-candidates that are true negatives = TN/(FN + TN). ^*a*^Number of species (or strains) per dataset (see **Table 1**). ^*b*^Each member of the filtered ortholog group have a specific greater than or less than protein sequence similarity. Note that there are no results for dataset containing 55 species with member sequences >70% and <99% (the processing for this dataset was terminated after 15 days). ^*c*^Time shown in hours (h), minutes (m), and seconds (s). Computer processing performed on a Linux cluster running Red Hat Enterprise Linux 7 (RHEL7) with the following specifications: 3.4 GHz Intel Xeon E5-2687W v2 (8 Cores) 25 MB L3 Cache (Max turbo 4.0 GHz, Min. 3.6 GHz), 32 GB 1866 MHz ECC DDR3-RAM (Quad Channel), 2x 2TB 7,200 RPM SATA III Hard Drives (Raid).*

**Supplementary Table S3** lists the predicted candidates, i.e., the true and false positive proteins for each species dataset and sequence similarity criteria (excluding hypothetical proteins). For the >70% and <95% similarity, the majority of the protein IDs identified as true positives are the same within each dataset, whereas the IDs mostly differ for the false positives. For example, there are 40, 38, and 40 true positives for the 16, 25, and 55 species datasets, respectively; of which 33 have the same IDs in all three datasets. Conversely, there are 3, 6, and 6 false positives for the 16, 25, and 55 species datasets; of which only one has the same ID in all three datasets. The candidate names from each dataset are listed side-by-side in **Supplementary Table S2** to highlight the consensus in the predictions. Overall, the 16 species dataset with >70% and <95% similarity delivers the best results given that it has the most true and the least false number of positives, i.e., has the best PPV with 93% (referred to henceforth as the elected dataset). The number of false negatives for the elected dataset compares favorably with the other species datasets, but the negative predictive value (NPV) is poor in comparison due to the substantially less number of true negatives used in the NPV computation, e.g., 27, 389, and 400 true negatives for the 16, 25, and 55 species datasets, respectively.

In this study, we also evaluated the McDonald–Kreitman test (MKtest) (McDonald and Kreitman, 1991), Tajima’s *D* (Tajima, 1989), and Wright’s fixation index (*F*_ST_) (Brown, 1970) to assess their suitability for the presented pipeline. Although these latter methods can be used to indicate positive selection, they were not designed for that purpose. The candidate predictive outcomes from MKtest, Tajima’s *D*, and *F*_ST_ using the same elected dataset were compared with the CODEML outcomes. **Supplementary Table S4** shows the predictive comparisons. Tajima’s *D*, *F*_ST_ and CODEML produced similar outcomes, but MKtest method, at least for the elected dataset, had substantially poorer predictive power.

**FIGURE 3:**
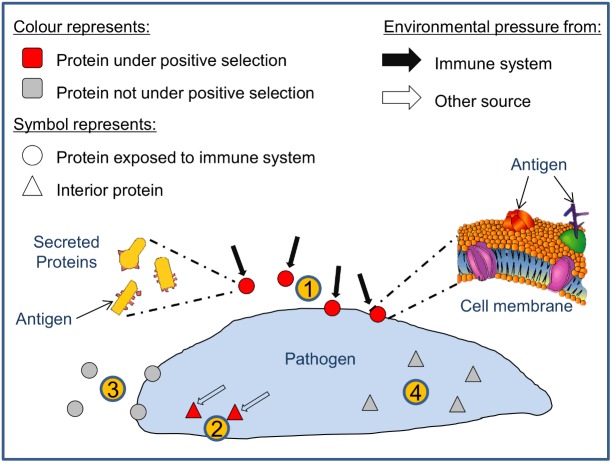
The four parasite protein classifications based on selection and subcellular location. Parasite proteins can theoretically be classified into four types: (1) proteins exposed to the immune system and under positive selection (i.e., potential vaccine candidates); (2) proteins not exposed but under positive selection; (3) proteins exposed but not under positive selection; and (4) proteins that are neither exposed nor under positive selection.

All pipeline results related to the elected dataset were compiled in **Supplementary Table S5**. This includes for each TGME49 member of the processed ortholog groups: the counts for the number of predicted positive selection sites by CODEML; the counts for the number of CODEML predicted sites associated with intermediate and high affinity peptides; and results from SignalP, TargetP, TMHMM; Phobius, WoLF PSORT, and Vacceed that provide indications of secreted or membrane-associated characteristics (i.e., potential exposure to the immune system). The results were categorized into four groups based on the sum of the number of positive sites > 95% *and* the number of positive sites > 99% posterior probability (i.e., the total significant positive sites count); and the Vacceed score (recorded in ‘*P* > 95 + 99%’ and ‘Vacceed_score’ columns in **Supplementary Table S5**. *P* > 95 + 99% referred to henceforth as the site count). Group one comprises those proteins predicted to be exposed to the immune system and containing positive selection sites, i.e., potential vaccine candidates (Vacceed score ≥ 0.5 and site count > 0), group two have proteins predicted to be not exposed but containing positive selection sites (Vacceed score < 0.5 and site count > 0), group three have proteins predicted to be exposed but not under positive selection (Vacceed score ≥ 0.5 and site count = 0); and group four proteins are neither exposed nor under positive selection (Vacceed score < 0.5 and site count = 0). **Figure 3** shows a schematic of the four groups, as classified by a protein’s selection and subcellular location status. Group one, the candidates (excluding hypothetical proteins), were ranked based on the number of consecutive positive selection sites on intermediate and/or high binding MHC I peptides (recorded in the ‘Consecutive PSS’ column in **Supplementary Table S5**). The top 10 ranked candidates are listed in **Table 5**. All 10 are target candidates. Furthermore, the program output values from SignalP, WoLF PSORT, TargetP, TMHMM, and Phobius support that all top 10 candidates are either secreted or membrane-associated.

**Table 5:** The top 10 predicted *Toxoplasma gondii* ME49 vaccine candidates for this study, i.e., proteins predicted to be exposed to the immune system, under positive selection, and contain consecutive positive selection sites on intermediate and/or high binding MHC I peptides.

Protein ID	Protein name^a^	No. of sites	No. of sig. sites	Consc. PSSs	Max. No. consecutive	Exposed probability	Reference
TGME49_227280	Dense granule protein GRA3	20	20	179	8	0.99	Craver and Knoll, 2007; Bontell et al., 2009; Rosenberg et al., 2009^∗^
TGME49_310780	Dense granule protein GRA4	82	21	153	8	0.92	Kur et al., 2009; Dziadek et al., 2012; Meng et al., 2013
TGME49_309330	SAG-related sequence SRS55F	63	18	107	5	0.82	Kim et al., 2007; Bontell et al., 2009; Wasmuth et al., 2012
TGME49_320190	SAG-related sequence SRS16B^b^	72	26	52	4	0.90	Kim et al., 2007; Wasmuth et al., 2012^∗^
TGME49_320200	SAG-related sequence SRS16A	39	13	41	3	0.94	Wasmuth et al., 2012; Hehl et al., 2015
TGME49_215775	Rhoptry protein ROP8	134	13	40	2	0.86	Parthasarathy et al., 2013; Zhang et al., 2016; Song et al., 2017
TGME49_214080	Toxofilin^c^	39	14	36	3	0.94	Bontell et al., 2009; Song et al., 2017^∗^
TGME49_205250	Rhoptry protein ROP18	87	11	35	3	0.98	Bontell et al., 2009; Qu et al., 2013; Behnke et al., 2015; Grzybowski et al., 2015; Zhang et al., 2016; Song et al., 2017^∗^
TGME49_238440	SAG-related sequence SRS22A	28	18	30	4	0.59	Hehl et al., 2015
TGME49_308090	Rhoptry protein ROP5	20	7	29	3	0.86	Bontell et al., 2009; Behnke et al., 2015; Chen et al., 2015; Grzybowski et al., 2015^∗^


*^*a*^Hypothetical proteins are not included in the list. ^*b*^Formally SRS9. ^*c*^Rhoptry protein. No. of sites = Total number of positive selection sites predicted by CODEML; No. of sig. sites = the number of positive sites > 95% + number of positive sites > 99% posterior probability as predicted by Bayes Empirical Bayes (BEB) analysis within CODEML; Consc. PSS = number of consecutive positive selection sites located on intermediate and/or high binding major histocompatibility complexes (MHC) class I peptides; Max. No. consecutive = maximum number of consecutive positive selection sites on a single MHCI peptide; Exposed probability = a score from 0 to 1 predicted by Vacceed that indicates the protein is likely to be a secreted or membrane-associated (a ‘1’ indicates ‘highly likely’); References = reference to a published study that supports the protein’s vaccine candidacy (a ‘^∗^’ supports positive selection status).*

**FIGURE 4:**
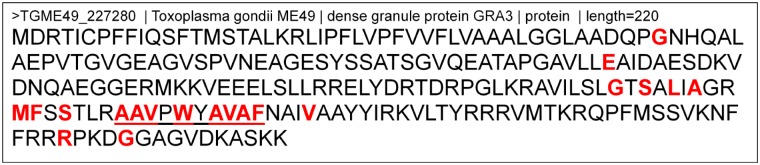
Location of predicted positive selection sites and one MHC-binding peptide on the dense granule protein GRA3 from *Toxoplasma gondii* strain ME49. This is the entire protein sequence of the dense granule protein GRA3. Letters highlighted in red are amino acid sites predicted to be under positive selection by CODEML (with a posterior probability > 99%). The letters underlined represent an encoded major histocompatibility complexes (MHC) binding peptide, i.e., a peptide (AAVPWYAVAF) was predicted to strongly bind to MHC Class I HLA-C^∗^03:02. This peptide of length 10 contains eight sites under positive selection.

The association between predicted binding peptides and positive selection sites were examined for the top 10 candidates. For example, GRA3 is predicted to contain 20 highly significant positive selection sites. All 20 of these positive selection sites are associated with 75 out of 304 MHC allele-peptide length combinations. That is, a site can be associated with more than one allele, e.g., the 144th GRA3 amino acid (letter ‘G’) is a positive selection site and is associated with HLA-A^∗^02:01 (peptide length 9), HLA-A^∗^02:06 (peptide length 10) and a further 23 other allele-peptide length combinations. A site can also be associated with one or more peptides, e.g., the 144th GRA3 amino acid is on the peptide ‘VILSLGTSA’ and ‘ILSLGTSAL’ that bind to HLA-A^∗^02:06 (peptide length 9). The GRA3 positive selection sites are spread on 78 different peptides. More than one site can be on the same peptide (referred in this study as consecutive sites). The maximum number of observed consecutive sites for GRA3 was eight and is illustrated in **Figure 4**.

**FIGURE 5:**
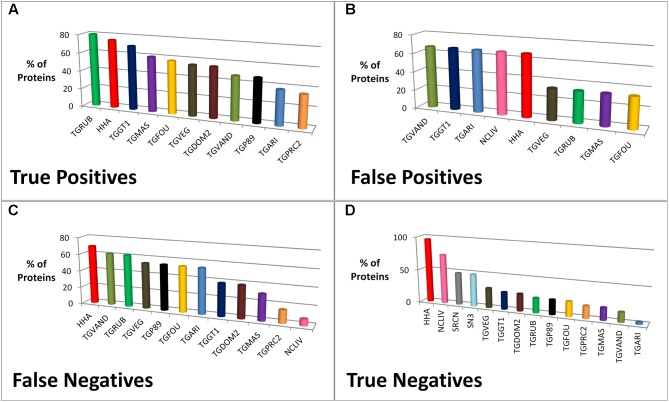
The percentage of proteins from a species or strain contributing to the prediction of *T. gondii* ME49 vaccine candidates (meanings of species abbreviations shown in **Table 1**). The graphs **(A–D)** illustrate the percentage of proteins from representative species or strains of the 16 species dataset contributing to ortholog groups. More specifically, it shows the collective contribution to recipient ortholog groups associated with the prediction outcomes (true positives, false positives, false negatives, and true negatives) when predicting vaccine candidates for *T. gondii* ME49. For example, in graph [A] 40 out of 143 ortholog groups contain a *T. gondii* ME49 protein that was correctly predicted as a candidate, i.e., a true positive. The species *Hammondia hammondi* strain H.H.34 (HHA) contributes a protein to 75% of ortholog groups containing a true positive protein, i.e., 30 of the 40 groups contain a HHA protein.

An analysis of the species type contributing members to the elected dataset ortholog groups was performed. For example, ROP8 (one of the top 10 candidates) has five members (proteins) in its ortholog group and in effect five species or strains (TGME49, TGGT1, TGRUB, TGVEG, and HHA) have contributed to the group. Each member of the group has a sequence similarity within the range 70–95%. **Figure 5** shows the species or strain contributions for the prediction outcomes (TP, FP, FN, and TN) when using the elected dataset. Only *T. gondii* RH (TogoCp) did not contribute to an ortholog group, but this species has only 26 known proteins. The species HHA contributes the most members to the ortholog groups (over 71%) and consequently is a major contributor to all four prediction outcomes. TGRUB is the largest (80%) and TGPRC2 is the smallest (35%) contributor to the ortholog groups that possess TP candidates; and all *Toxoplasma* strains (except TogoCp) and HHA make a contribution. Conversely, NCLIV, SN3, and SRCN make no contributions to TP predictions, but contribute more than all the *Toxoplasma* strains to TN predictions (74, 48, and 48%, respectively). When using the 25 or 55 species datasets, the contributing species to the TP predictions were exactly the same as the 16 species contributions, although in slightly different proportions. However, the contributions to the other prediction outcomes (FP, FN, and TN) were notably different. **Supplementary Table S2** shows the comparison of the species contributions between the 16, 25, and 55 species datasets.

Our pipeline was also tested with other target pathogens; namely, *T. gondii* p89 (TGP89), HHA, and NCLIV. TGP89 is one of the most distantly related strains to *T. gondii* based on haplogroups (Su et al., 2012); *H. hammondi* is the closest extant relative to *T. gondii* (Jenkins et al., 1999);and *N. caninum* is morphologically and developmentally similar to *T. gondii* (Bjerkas and Dubey, 1991). *Hammondia* and *Neospora* are still significantly different in that they have never been found to infect humans (Barratt et al., 2010; Walzer et al., 2013). Both TGP89 and HHA have appropriate protein annotation to identify target candidates and evaluate pipeline prediction outcomes. The PPVs for TGP89 and HHA candidates were 86 and 68%, respectively, using the 16 species dataset with >70 and <95% sequence similarity. NCLIV, in contrast, has limited annotation both in ToxoDB and National Center for Biotechnology Information (NCBI) to use exclusively for evaluation (one putative dense-granule antigen DG32, five putative microneme proteins, five SAG related proteins, and no rhoptry proteins). To assist in evaluating NCLIV predicted candidates, the protein description from their closest homolog was used. Despite this, 15 of the 45 candidates remained hypothetical, and no target candidates were identified. We also used the top 10 *Toxoplasma* candidate sequences as BLASTP queries to find the closest *Neospora* homolog. This showed, for example, that there was no NCLIV homolog for GRA3, NCLIV_054830 (unspecified product) was the closest homolog to GRA4 with 37% similarity, NCLIV_001970 (unspecified product) was closest to ROP 8 with 47%, NCLIV_051340 (putative toxofilin) to toxofilin with 34%, NCLIV_060730 (putative ROP5) to ROP18 with 30%, and NCLIV_060730 to ROP5 with 52% similarity. The net finding was that these latter NCLIV proteins are too distantly related to contribute to the TGME49 candidate ortholog groups. Additionally, the former NCLIV sequences were used as BLASTP queries to find the closest homolog species. For example, NCLIV_054830 was closest to TGDOM2_310780 (GRA4) with 31%, and NCLIV_001970 was closest to NCLIV_001950 (Rhoptry protein ROP7, related) with 53% similarity. The net finding, when running our pipeline with NCLIV as the target species, was that no ortholog groups were created for these latter NCLIV sequences. This is because there are no known homolog sequences with greater than 70% similarity.

The predicted candidates for TGP89, HHA, and NCLIV are listed in **Supplementary Table S6** along with predictions for *T. gondii* GT1 (PPV = 98%), *T. gondii* MAS (PPV = 91%), *T. gondii* RUB (PPV = 95%), and *T. gondii* VEG (PPV = 94%). ROP18, ROP16, and toxofilin were predicted candidates in all *T. gondii* strains.

## Discussion

The aim of this study was to evaluate a positive selection detection method in its contributing capacity to identify potential protein vaccine candidates, given thousands of anonymous protein sequences from a pathogenic protozoan. Data from the protozoan *T. gondii* ME49 were chosen for the evaluation because *Toxoplasma* is a model system for the phylum Apicomplexa and the ME49 genome sequence is the primary *T. gondii* reference. Furthermore, the protein name annotation is comparatively better than other apicomplexan species.

There is no known subunit vaccine against *Toxoplasma* or indeed against any apicomplexan species. Hence this study had no definitive target protein-type to validate our methodology. However, SAG-related, GRA, MIC, and ROP proteins have received the most focus in recombinant/subunit vaccine studies and are therefore judged to be suitable target candidates. There are 244 proteins that were classified target candidates based on their protein name. Nevertheless, it is important to emphasize that the true immunogenic efficacy for the majority of these target candidates is unknown. Our premise under evaluation here is that a protein naturally exposed to the immune system and containing epitopes under positive selection will make a more worthy vaccine candidate for laboratory testing than a protein without these characteristics (Doolan, 2011; Jones, 2012; Donati and Rappuoli, 2013; Oprea and Antohe, 2013). We believe that in identifying the target candidates, it provides a homing mechanism to the worthy candidates. There are 8263 TGME49 proteins and the probability of randomly selecting a target candidate is 2.9% (244/8263).

We created a *high-throughput* pipeline to identify the target candidates, given the high number of proteins and the impracticality of investigating protein candidacy on a case-by-case basis. Our pipeline used freely available, standalone bioinformatic programs. A considerable drawback to an automated process, such as a pipeline, is that a generic set of parameters and threshold values are applied to *all* data. For example, each program in our pipeline has tens or hundreds of changeable parameter settings (especially RAxML and CODEML) that have varying degrees of impact on results. Similarly, different threshold values can be set to classify output data, e.g., a Vacceed score ≥ 0.5 denotes an immune-system exposed protein and a site count > 0 denotes protein under positive selection. Taken together, there are potentially hundreds of user-defined combinations of parameters and thresholds with the net effect of fluctuating prediction outcomes (TPs, FPs, FNs, and TNs). The desired intention of our pipeline was therefore to favor combinations that greatly increased the probability of identifying target candidates (i.e., a high PPV outcome) at the possible detriment to the NPV.

There are several recognized methods to detect positive selection (reviewed in Wollstein and Stephan, 2015) and freely available programs to apply these methods. The dN/dS ratio method implemented in the program CODEML was chosen for our pipeline. CODEML has the important functionality to identify positive selection occurring at individual amino acids (sites), unlike other methods evaluated in this study. This was important because we specifically sought to ascertain if predicted epitopes contained positive selection sites.

At the heart of the positive selection detection method performed by CODEML is the estimation of non-synonymous and synonymous distances from a coding sequence alignment of orthologous genes. A key component to the success of this method is the creation of appropriate ortholog groups. The main factor affecting appropriateness is how closely or distantly related are the sequences of the group members. It was unclear at the onset of this study, what the upper and lower sequence similarity thresholds should be for group membership to provide optimum predictive power that ultimately detects the greatest number of target candidates. To address this uncertainty, we tested our pipeline with varying similarity thresholds, and varying numbers and types of contributing species. In effect, the testing increased or decreased the number of observed sequence changes in accordance with the introduction or removal of sequences, such that these test scenarios either increased or decreased CODEML’s power to accurately estimate dN and dS, e.g., too few observed changes, too little power. Also, CODEML’s intended use is to observe these changes in protein coding sequences from *divergent* species. Whether the introduced sequences are actually from *divergent* species is at the onus of the CODEML user. That is, all ortholog group sequences are treated exactly the same during the codon-based alignment and phylogenetic tree creation, irrespective of the sequences’ origin.

It is arguable whether observed changes between *T. gondii* strain sequences in the ortholog groups represent fixation events along independent lineages (i.e., substitutions in diverging species) or polymorphisms segregating in a single population (i.e., mutations within the strains). Furthermore, fixed substitutions are expected to occur over long time-scales (Kryazhimskiy and Plotkin, 2008) and it is difficult to know the appropriate time-scale associated with each contributing strain. A study applying clustering methods to 950 isolates collected from around the world identified 15 haplogroups that collectively define six major clades (A–F) in *T. gondii* (Su et al., 2012). In our opinion this latter study suggests that some *T. gondii* strains are diverging more than others. That is, although all isolates of the genus *Toxoplasma* have been classified a single species, their global and isolated distribution has allowed for independently evolving strains. Clade ‘A’ contains the strains TGGT1 and TGFOU; clade ‘B’ contains TGMAS; ‘C’ contains TGVEG; ‘D’ TGME49 and TGARI; ‘E’ TGP89; and ‘F’ contains TGRUB and TGVAND. Clade ‘D’ has the highest level of divergence from other clades (Su et al., 2012) and typically clade ‘D’ strains have a closer relationship to C than A such that the relationships are D - > C - > A - > B - > F - > E. The similarity relationships between the members of the top 10 ortholog groups only loosely follow the clade relationships, which indicate that there may be a typical phylogenetic relationship between the *T. gondii* strains but not their proteins. Hence, creating a phylogenetic tree from protein-coding sequences associated with *each* ortholog group is deemed a better solution than creating one phylogenetic tree from the genome sequences of the strains.

The results clearly showed, at least for *T. gondii* ME49, that there is an ideal ‘Goldilocks’ range for the sequence similarity thresholds, i.e., >70 and <95. The same thresholds are supported by another study that used CODEML with *Plasmodium* parasites (Nygaard et al., 2010). Thresholds greater than 95 possibly introduce more polymorphism observed sequence differences. Our testing also showed that including more species related to *T. gondii ME49* (i.e., those in the 25 and 55 species datasets), with the potential of having more sequences within the range >70 and <95 contributing to the group, did not improve the predictive power.

The best PPV (93%) was achieved using the 16 species dataset with the Goldilocks similarity range. Our approach worked effectively in the sense that out of 53 target candidates represented in 83 ortholog groups, 40 were correctly identified (130 groups were processed but 47 contained ME49 hypothetical proteins). It is reasonable to assume that some of the CODEML predictions are incorrect, given the inherent deficiencies in all programs *per se*. We judge, however, that a protein predicted to contain many significant positive sites will likely have an unknown percentage to be true; whereas, chances for a true prediction are less likely with only a few predicted significant sites. Furthermore, a protein predicted to contain a positive selection site on a functional region (e.g., encoding an epitope) is weighted more highly here than on a non-functional region. Given the conceivably high number of epitopes encoded in a protein sequence, the chances of randomly selecting a site on an epitope is potentially high. However, these chances incrementally reduce the more sites that are predicted on the same epitope. The 40 candidates were ranked on the number of consecutive positive selection sites on intermediate and/or high binding MHC I peptides. **Table 5** shows the top 10 ranked candidates. The highest ranked are deemed the most promising. A considerable number of publications support the top 10, although published vaccine candidacy evidence for ‘SAG-related sequence SRS’ proteins was difficult to find. This difficulty was also enhanced due to protein name changes, i.e., both new and historical publications use different names for the same SRS proteins, e.g., SRS29B or the original SAG1, and SR16B or the original SRS9.

An unknown element in our study is how many of the 244 target candidates will truly contribute toward protective immunity. This means that it is unclear as to what extent our approach has missed true candidates, i.e., only 53 out of 244 target candidates were represented in the 130 ortholog groups. Most of the target candidates have no published vaccine candidacy evidence, especially the ‘SAG-related sequence SRS’ or ‘Toxoplasma gondii family’ proteins. The conundrum is whether the lack of evidence indicates that these proteins are not worthy or are unexploited candidates for vaccine candidacy investigation. Despite this, there are clearly some target candidates with published evidence missed by our approach; for instance, GRA2, GRA5, GRA7, ROP7, and SRS29B (Kur et al., 2009; Dziadek et al., 2012). Most of the missed target candidates are captured by increasing the upper similarity threshold to 99% (see **Supplementary Table S3**), but this is at the expense of a substantial increase in false positives. The proteins GRA9, GRA10, GRA12, ROP1, ROP6, ROP7, and all MICs are not captured as candidates because the similarity between most of their respective ortholog members is >99% and the minimum five member requirement was not fulfilled (ROP2A and ROP4 were not processed due to invalid sequences – see **Supplementary Table S2**).

Our testing has shown that CODEML and Vacceed work satisfactorily in distinguishing immune system-exposed proteins under positive selection, i.e., our approach can capture the majority of target candidates when given an appropriate ortholog group with valid sequences, but obviously fails if there is no ortholog group to process. Furthermore, the described approach can be adapted for other apicomplexan parasite species or strains with appropriate data as supported by the TGP89 and HHA prediction results.

The high levels of evolved genetic variation for the target candidates present a major challenge for the development of an effective vaccine. This is because an immune response generated against one allele might not be effective against a different allele expressed by a parasite of the same species (Elsheikha and Mansfield, 2004; MacHugh et al., 2011). To help address this challenge we propose that all protein members of a candidate’s ortholog group are potential candidates, based on common conjecture that proteins with similar sequences are likely to have similar functions. This proposal is supported by the fact many of the same candidates were predicted irrespective of the *Toxoplasma* strain chosen as the target species (see **Supplementary Table S6**). For example, the TGME49 ROP18 ortholog group contains TGGT1_205250 as a member. TGGT1_205250 was predicted as a candidate when *T. gondii* GT1 (TGGT1) was the chosen target species. In fact, ROP18, ROP16, and toxofilin were predicted as a candidate for all strains. This is important because of the need for vaccines to contain two or more distinct antigens, or two or more alleles of the same antigen to protect against multiple species and diverse strains, i.e., multivalent vaccines that represent the majority of the genetic diversity of candidate antigens (Barry and Amott, 2014).

Our approach was unable to identify target candidates for NCLIV, although its limited annotation did make evaluation difficult. A possible reason for this disappointing result was the absences of a clear outgroup species and diverging sequences afforded by other *Neospora* strains. For example, HHA was the outgroup species (i.e., the most distantly related species) for 29 out of the 40 true positive candidates in the TGME49 elected dataset. Moreover, the majority of TGME49 ortholog groups would not have been created without the membership contributions from additional *Toxoplasma* strains, i.e., there are not enough available sequences from other species with greater than 70% similarity. *H. heydorni* is the sister taxon to *N. caninum* (Dubey et al., 2002) in a similar manner to how *H. hammondi* is the sister group to *T. gondii*. We expect improved NCLIV results from our pipeline when quality *H. heydorni* and additional *N. caninum* strain sequences become available.

A challenge as highlighted by missed candidates is in determining pipeline threshold values, which in effect governs the creation of an ortholog group. A high throughput solution is sought to either weed out false positives based on additional selection criteria when using high similarity thresholds or to judiciously vary the ortholog membership requirements for each protein with the goal of capturing the maximum number of worthy candidates. Nevertheless, what is encouraging is that ROP5 and ROP18 are virulence determinants (Taylor et al., 2006; Walzer et al., 2013) and our pipeline classified both as top 10 candidates. The chance of randomly classifying a ROP protein was 0.2% given 8263 anonymous sequences.

Possibly the utmost important task of an *in silico* approach to vaccine discovery is to distinguish antigenic from non-antigenic pathogen proteins. Most *in silico* studies (Pizza et al., 2000; Ross et al., 2001; Wizemann et al., 2001; Montigiani et al., 2002; Doytchinova and Flower, 2007; Donati and Rappuoli, 2013) use a filtering approach based on specific protein characteristics but mainly protein localization (e.g., secretory, outer-membrane). More recent studies (Doytchinova and Flower, 2007; Bowman et al., 2011; Goodswen et al., 2013) have incorporated machine learning algorithms into the reverse vaccinology methodology. It is our opinion that these approaches should not be used in isolation. The best strategy is to strive for a consensus of predicted antigens from several approaches. We conclude that an approach to classify those proteins naturally exposed to the immune system and containing epitopes under positive selection, such as the one presented here, is a valuable addition to other *in silico* approaches to identify vaccines candidates worthy of laboratory validation.

## Author Contributions

SG, PK, and JE contributed to the experimental design and production of the manuscript. SG performed all the analyses described.

## Conflict of Interest Statement

The authors declare that the research was conducted in the absence of any commercial or financial relationships that could be construed as a potential conflict of interest.
