# Supplementary material for: A Gene-Based Positive Selection Detection Approach to Identify Vaccine Candidates Using Toxoplasma gondii as a Test Case Protozoan Pathogen
Source: Front Genet. 2018 Aug 20;9:332. doi: 10.3389/fgene.2018.00332 (PMC6109633; doi:10.3389/fgene.2018.00332)
Supplement: Supplementary file 1 [file Table_1.pdf]

*Supplementary Material*

# A gene-based positive selection detection approach to identify vaccine candidates using *Toxoplasma gondii* as a test case protozoan pathogen

Stephen J. Goodswen, Paul J. Kennedy, John T. Ellis*

**Correspondence:** John Ellis: John.Ellis@uts.edu.au

**Supplementary Table S1:**Command-line syntax for programs used in study

| Program[a] | Command-line syntax with parameters[b] |
|---|---|
| BLASTP | blastp -query prot_Toxoplasma_ME49.fasta -subject prot_Toxoplasma_FOU.fasta -outfmt "10 evalue qseqid qlen sseqid slen bitscore score pident nident mismatches positive" -max_target_seqs 5 -out blastp_Toxoplasma_ME49 |
| Clustal Omega | clustalo -i mrna_sequences.txt -o clustal_output --force |
| PAL2NAL | pal2nal.pl clustal_output mRNA_sequences.fasta -output paml -nogap > pal2nal_output |
| RAxML | raxmlHPC-PTHREADS -f a -x 12345 -p 12345 -# 100 -m GTRGAMMA -s pal2nal_output -n raxml_output -w out_dir |
| CODEML | codeml control_file.ctl > output_file |
| predict_binding.py | predict_binding.py IEDB_recommended HLA-A*23:01 9 input.fasta |
| MKtest | GENOME.class <- set.populations(GENOME.class,list(within_pop,between_pop))<br>GENOME.class <- MKT(GENOME.class,do.fisher.test=TRUE)<br>results <- get.MKT(GENOME.class)<br>neutrality.index <- results[1,"neutrality.index"] |
| Tajima's D | GENOME.class <- neutrality.stats(GENOME.class)<br>tajima_result <- GENOME.class@Tajima.D |
| $F_{ST}$ | GENOME.class <- F_ST.stats(GENOME.class)<br>get.F_ST (GENOME.class,mode="nucleotide")<br>Results <- get.diversity (GENOME.class)<br>fst_nucelotide <- results[1,"nucleotide.F_ST "] |

[a]MKtest = the McDonald–Kreitman test, $F_{ST}$ = Fixation index. MKtest, Tajima's D and $F_{ST}$ methods are performed using R functions from PopGenome (https://cran.r-project.org/web/packages/PopGenome/index.html).

[b]-f a = selects the algorithm to be 'rapid Bootstrap analysis and search for best scoring ML tree in one program run'; -x = rapid Bootstrap random number seed; -p = parsimony random seed; -# = number of runs; -m GTRGAMMA = generalised time reversible (GTR) GAMMA substitution model.
