# Supplementary material for: A Gene-Based Positive Selection Detection Approach to Identify Vaccine Candidates Using Toxoplasma gondii as a Test Case Protozoan Pathogen
Source: Front Genet. 2018 Aug 20;9:332. doi: 10.3389/fgene.2018.00332 (PMC6109633; doi:10.3389/fgene.2018.00332)
Supplement: Supplementary file 4 [file Table_4.pdf]

Supplementary Material

A gene-based positive selection detection approach to identify vaccine candidates using *Toxoplasma gondii* as a test case protozoan pathogen

Stephen J. Goodswen, Paul J. Kennedy, John T. Ellis*

Correspondence: John Ellis: John.Ellis@uts.edu.au

Supplementary Table S4: Comparisons between predicted outcomes from different positive selection detection methods when predicting target candidates for *Toxoplasma gondii* ME49

Detection method[a]	TP	FP	FN	TN	SP (%)	SN (%)	PPV (%)	NPV (%)
Tajima D	35	3	15	27	70	90	92	64
MKtest	17	4	33	25	34	86	81	43
F_{ST}	37	2	15	29	71	94	95	66
CODEML	40	3	13	27	75	90	93	68

[a]Positive selection detection method: for Tajima's D, positive selection = a negative value; for the MKtest, positive selection = neutrality index (NI) < 1; for F_{ST}, positive selection = high values (> or = 0.7 used here); for CODEML, positive selection = significant positive selection sites count > 0. Note that the processing of 3 proteins for Tajima D and 4 for MKtest did not result in a valid score (these proteins were ignored from outcome calculations).

A predicted candidate is one that matches the positive selection threshold for the detection method *and* has a Vacceed score greater than or equal to 0.5. The target candidates are proteins containing either the words 'dense granule protein GRA', 'microneme protein MIC', 'rhoptry protein ROP', 'SAG-related sequence SRS', or 'Toxoplasma gondii family' as part of their protein name (these proteins are naturally exposed to the immune system and expected to have sites under positive selection). TP = true positives = number of correctly predicted target candidates, FP = false positives = number of exposed proteins under positive selection but not recognised as target candidates; FN =false negatives = number of target candidates incorrectly predicted to be non-exposed and/or under negative or neutral selection; TN = true negatives = number of proteins correctly predicted to be non-exposed and under negative or neutral selection; SN = sensitivity = % of target candidates correctly predicted = TP /(TP + FN), SP = % of non-candidates correctly predicted = TN/(FP + TN); PPV = positive predictive value = % of target candidates that are true positives = TP/(TP + FP); NPV = negative predictive value = % of non-candidates that are true negatives = TN/(FN + TN).
